# Neurosyphilis: An Unresolved Case of Meningitis

**DOI:** 10.1155/2015/634259

**Published:** 2015-05-14

**Authors:** Shagufta Ahsan, Joesph Burrascano

**Affiliations:** ^1^Saint Michael's Medical Center, 111 Central Avenue, Newark, NJ 07102, USA; ^2^International Lyme and Associated Diseases Society, Bethesda, MD 20827-1461, USA

## Abstract

Neurosyphilis can cause both symptomatic and asymptomatic meningitis. However the epidemiology of modern neurosyphilis is not well defined because of the paucity of population-based data. The majority of neurosyphilis cases have been reported in HIV-infected patients. Here we present a case of early neurosyphilis/symptomatic syphilitic meningitis in a non-HIV patient who presented with rash but was mistakenly treated for early latent or secondary syphilis. Syphilis presenting with a skin rash and an extremely high RPR titer could indicate CNS infection rather than simply secondary syphilis because rash is a nonspecific manifestation of disseminated infection. Given the effectiveness of penicillin therapy, why is the rate of syphilis continuing to increase? Is it due to a failure of prevention or could it be also because of failure to diagnose and treat syphilis adequately, as in this case?

## 1. Introduction

Neurosyphilis can cause both symptomatic and asymptomatic meningitis. However the epidemiology of modern neurosyphilis is not well defined because of the paucity of population-based data [1]. The majority of neurosyphilis cases have been reported in HIV-infected patients. Here we present a case of early neurosyphilis/symptomatic syphilitic meningitis in a non-HIV patient who presented with rash but was mistakenly treated for early latent or secondary syphilis. Syphilis presenting with a skin rash and an extremely high RPR titer could indicate CNS infection rather than simply secondary syphilis because rash is a nonspecific manifestation of disseminated infection.

Given the effectiveness of penicillin therapy, why is the rate of syphilis continuing to increase? Is it due to a failure of prevention [1] or could it be also because of failure to diagnose and treat syphilis adequately, as in this case?

## 2. Case Description

A 24-year-old female was seen in an emergency room of a hospital in Brooklyn, New York, for a severe and persistent headache for the prior two weeks. She has had history of chronic intermittent migraine headache. However, this headache was different in that pain involved the whole scalp including the forehead and was constant and severe in nature. Her headache was associated with significant dizziness and vertigo but she did not report any fever, nausea, vomiting, generalized or focal weakness, or dysarthria. Patient had weight loss of fifteen pounds over several months. No oncologic work up and endocrine evaluation were done. She traveled to Aruba 2 years prior and had no history of tuberculosis exposure at that time. She did not recall tick bites nor engage in outdoor activities recently. The patient has not been on any medications and does not have a history of illicit drug use.

Examination revealed a normocephalic, atraumatic obese woman. Sinus tenderness was not present and ENT exam was normal. Pupils were equal and reactive to light. Patient was awake, alert, and oriented. Neurological examination was non focal with full motor strength and no sensory loss. Moderate neck stiffness was present, and no meningeal signs were noted. There was no palpable hepatosplenomegaly or lymphadenopathy. Genital examination showed no vesicles, rashes, or ulcers. Patient was afebrile.

## 3. Significant Past Medical History

Patient is heterosexual but denies being sexually active for the last two years. She has a history of sexual abuse and two sexually transmitted diseases (STDs), genital herpes and syphilis. The genital herpes were diagnosed in October 2010 and treated with oral acyclovir. Since then, there have not been any herpes recurrences. Subsequently in September 2011 the patient presented with a rash that was diagnosed as secondary syphilis. The rash was present only on the palms and was not generalized. She had an extremely high RPR titer of 1 : 500. She was given 1.6 million units of benzathine PCN G intramuscularly. The rash resolved in few weeks. Unfortunately her rash recurred on the left hand on April 2012 (seven months after treatment). At this time the RPR titer was 1 : 64. This time 2.4 million units of benzathine penicillin penicillin given intramuscularly. The rash resolved in one to two days. Follow-up RPR titer in May, 2012, was 1 : 16. It was thought that treatment was successful and further RPR follow-up was not done. During the whole period of this illness, the patient continued to have headaches on and off, and again the nature of this pain was somewhat different from that of her usual migraine headache.

## 4. Evaluation and Treatment

In January 2013 the patient presented with dizziness, a persistent headache of two weeks duration and moderate neck stiffness, and was admitted to the hospital. Her serum VDRL titer was 1 : 64. HIV rapid test was nonreactive. CT scan of head without contrast was normal. Lumbar puncture was done for the first time as a work up of subacute meningitis in light of the patient's history of syphilis. CSF analysis showed CSF WBC 18, lymphocytes 94%, mononuclear cells 6%, protein 35, and glucose 44. CSF cryptococcal Ag was negative and quantitative CSF VDRL was reactive at 1 : 16. CSF culture showed no growth.

Thus the final diagnosis for this case was early symptomatic neurosyphilis or syphilitic meningitis, which would explain the persistent headache, vertigo, and recurrence of rash secondary to inadequate prior treatment. PCN G, 4 million units intravenously every four hours, was started as a treatment for symptomatic syphilitic meningitis. Subsequently the patient's symptoms improved in one to two days and resolved completely in 4 to 5 days. Her symptoms of dizziness and nonmigraine headache have not recurred and the palmar rash never returned during her hospital stay. While patient was discharged she was asked to follow up with infectious diseases doctor's office, but patient refused to follow up.

## 5. Discussion

The clinical manifestations are traditionally divided into incubating, early infectious primary and secondary stages lasting 4 years, often marked with periods of symptoms and signs, and latency, which progresses to a late or tertiary stage of tissue destruction in up to a third of untreated patients [2].

### 5.1. Incubating Syphilis

The median incubation period before clinical manifestations is 21 days (range from 3 to 90 days). Left untreated, at least two thirds of patients spontaneously clear the infection, and the remaining third will clinically progress to late syphilis in 5 to 30 or more years [2].

### 5.2. Primary Syphilis

Primary syphilis presents 1 week to 3 months (median, 21 days) after exposure with a painless lesion, a chancre, at the site of inoculation and nontender regional lymphadenopathy. The lesion starts as a papule and rapidly forms an ulcer that is typically indurated, nonexudative with a clean base. Primary lesions are most commonly found on the external genitalia but can develop on any site of exposure including the perineum, cervix, anus, rectum, lips, oropharynx, and hands. Multiple chancres can occur, especially in persons who are immune suppressed as those coinfected with HIV.

### 5.3. Secondary Syphilis

**Figure 1:**
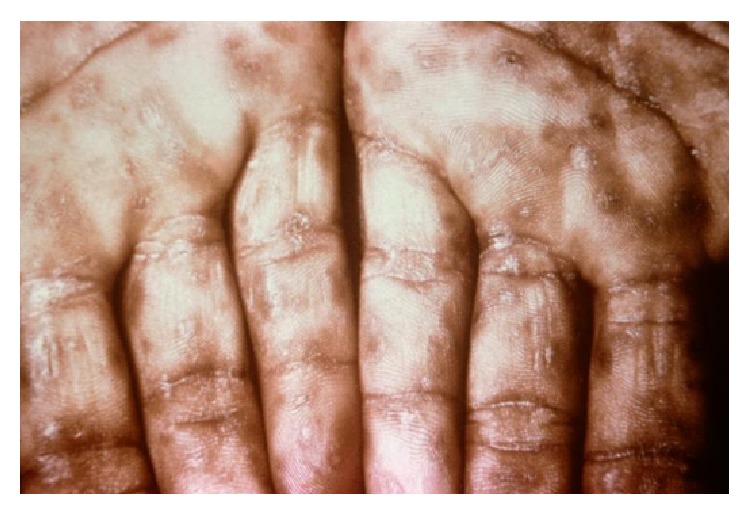
An example of secondary syphilis provided by the CDC: a close-up view demonstrating keratotic lesions on the palms.

The term secondary (disseminated) syphilis is used to describe the clinically most florid stage of infection; it results from multiplication and wide dissemination of spirochete and lasts until a sufficient host response develops to exert some immune control over the spirochete (Figure 1). It usually begins 2 to 8 weeks after the appearance of a chancre, but this period is variable. In some cases the primary chancre may be present.

The manifestations of secondary syphilis are widespread and protean. The classic and most commonly recognized lesions involve the skin. The cutaneous manifestations of secondary syphilis are diverse. The classic exanthem of secondary syphilis is a diffuse maculopapular rash that often, but not always, involves the palms and soles and scrotum. However, the rash can also be papular, annular, or pustule and can have a fine overlying scale. Other mucocutaneous manifestations include (1) Condylomata lata (moist heaped-up broad plaques found in intertriginous areas, such as the perianal area, vulva, and inner thighs; (2) mucous patches (gray, superficial erosions or plaques on the buccal mucosa and tongue, under the prepuce and on the inner labia; (3) split papules (fissured, nodular lesions at the angle of the lips and in the nasolabial folds); and (4) patchy alopecia (thinning of hair, eyebrows, and beard caused by syphilitic involvement of the hair follicle).

The cutaneous lesions of syphilis, particularly the nonkeratinized mucocutaneous lesions (Condylomata lata and mucous patches) contain large concentrations of spirochetes and are highly infectious.

The CNS becomes involved in up to 40% of patients as a result of seeding during the inevitable early spirochetemia. It may be asymptomatic or may manifest as acute symptomatic aseptic meningitis in 1 to 2% of patients [2]. Because of the omnipresent spirochetemia almost any organ of the body can be involved. The diverse manifestations of secondary syphilis earn it the name “the great imitator” [3].

### 5.4. Latent Syphilis

Without treatment, the manifestations of secondary syphilis generally resolve within a few weeks. The disease then enters a latent phase, characterized by a lack of clinical signs of syphilis, but positive serologic tests. However, it does not imply a lack of progression of disease only that clinical signs and symptoms are absent. Early latent syphilis distinguishes the period (first 4 years) during which a clinical relapse may occur and, therefore, the patient is infectious. Ninety percent of the relapses occur in the first year, and each recurring episode is less florid. Muco cutaneous relapses are the most common.

Late latent syphilis, at least in immunocompetent patients, is associated with host resistance to reinfection and to infectious relapse. However, a pregnant woman with late latent syphilis can infect her fetus in utero, and an infection can sometimes be transmitted via transfused contaminated blood.

### 5.5. Late Syphilis

Late syphilis (tertiary syphilis) is a slowly progressive destructive inflammatory disease that can affect any organ in the body to produce clinical illness 5 to 30 or more years after the initial infection. The three most common manifestations of tertiary disease are (1) neurologic/neurosyphilis, (2) cardiovascular syphilis, and (3) gummatous (or late benign) syphilis.

Late neurologic complications of syphilis, as described in Table 1 [2], present after long periods of latency, are caused by meningovascular and/or parenchymal damage.

### 5.6. Cardiovascular Syphilis

Endarteritis of the vasa vasorum of the aorta can lead to aortitis and aneurysm formation. This usually involves the ascending aorta, which in turn can cause dilatation of the aortic ring, aortic regurgitation, or ascending aortic aneurysm. Following the ascending aorta, the transverse aorta and then the descending arch are the next most common sites involved. Chronic inflammation of the coronary arteries can lead to narrowing and stenosis of the coronary ostia, which can ultimately lead to myocardial ischemia, infarction, and congestive heart failure [3].

### 5.7. Gummatous (or Late Benign) Syphilis

Gummatous disease is extremely uncommon and is characterized by indolent destructive lesions of the skin, soft tissue, and bony structures. Visceral organs and the CNS can also be involved [3].

## 6. How Common Is Early Syphilitic Meningitis in a Non-HIV Patient?

Early neurosyphilis involves diffuse inflammation of the meninges resulting in signs and symptoms of meningitis which can include headache, photophobia, nausea, vomiting, cranial nerve palsies, and occasionally seizures. Studies have shown that a significant number of cases of syphilitic meningitis occurred after inadequate treatment of early syphilis. The above case likely represents early neurologic involvement rather than inadequate dosing, as the patient complained of headache from the very beginning.

In the pre-HIV penicillin era, acute syphilitic meningitis was extremely rare suggesting that the combination of a robust immune system and an adequate dose of benzathine penicillin G was enough to control early CNS infection. This changed, however, in the HIV era when cases of acute syphilitic meningitis in penicillin treated and untreated patients with early syphilis were reported. Indeed, in some reports, early symptomatic neurosyphilis was the primary neurologic manifestation of* T. pallidum* infection in HIV-infected persons.

What could be the reasons why a previously healthy person with an apparently intact immune system, as in the case presented here, develops early symptomatic neurosyphilis? This needs to be further studied. A recently published study suggests that nontreponemal titer fluctuations in persons with HIV may reflect the influence of factors unrelated to syphilis diseases activity [4]. However, in a non-HIV patient with syphilis, any significant RPR variation (i.e., increase in RPR titers > 1 : 4) may reflect active disease, whether or not the patient had been treated.

In conclusion, even without HIV infection or immunodeficiency, neurosyphilis should always be a diagnostic consideration in early stage syphilis when initial serum RPR titers are 1 : 32 or higher [2].

*Management of Neurosyphilis*. Syphilis is caused by infection due to* Treponema pallidum* subspecies pallidum, a spirochete that cannot be visualized by light microscopy and cannot be cultured. Depending on the stage of infection, the diagnosis of syphilis is established by documentation of reactive serum nontreponemal tests (rapid plasma reagin {RPR} test or venereal disease research laboratory {VDRL test}) and treponemal tests (*T. pallidum* particle agglutination assay {TPPA} or fluorescent treponemal antibody-absorption {FTA-ABS} test) with or without characteristic symptoms or signs of syphilis.

When to include lumbar puncture to rule out syphilitic meningitis or neurosyphilis.

Because* T. pallidum* cannot be cultured, diagnosis of neurosyphilis relies on the following indirect measures: elevated CSF WBC count or protein concentration or reactivity of the CSF VDRL test. A reactive CSF VDRL test is considered to be diagnostic of neurosyphilis, but depending on the criteria used to define neurosyphilis, this test may be nonreactive in patients with neurosyphilis [4]. Thus, a positive VDRL establishes a diagnosis of neurosyphilis, but a negative VDRL does not exclude it. Another way to diagnose neurosyphilis is CSF FTA-ABS test which has high sensitivity, but low specificity because of high false positive rate. The CSF FTA-ABS can be used to exclude neurosyphilis in at-risk patients with an abnormal CSF and a negative CSF VDRL [3]. However, when diagnosing early neurosyphilis, the serum nontreponemal test (e.g., VDRL) is almost always positive. When diagnosing late neurosyphilis, the serum nontreponemal tests may be negative in up to 30% of persons, if clinical suspicion for late neurosyphilis is high; a treponemal test should be obtained even when the nonspecific lipoidal test is negative.

*How to Follow Up a Case of Neurosyphilis*. For early syphilis a fourfold decline in titers is expected 6–12 months after therapy. For late stage disease, a decline is expected 12–24 months after therapy. Serologic response, however, does not necessarily imply a microbiologic cure. The relationship between serology and microbiology is unclear [1].

Lack of serologic response may be due to either treatment failure or reinfection. The distinction between these two events is very difficult to make given the challenges of obtaining reliable information from persons on their sexual behaviors.

Follow-up of titers is very important as treatment failure can occur with any regimen. However, assessing response to treatment frequently is difficult, and definitive criteria for cure or failure have not been established. In addition, nontreponemal test titers might decline more slowly for persons who previously have had syphilis. Clinical and serologic evaluation should be performed 6 months and 12 months after treatment; more frequent evaluation might be prudent if follow-up is uncertain.

Patients who have signs and symptoms that persist or recur or who have a sustained fourfold increase in nontreponemal test titer (i.e., compared with the maximum or baseline titer at the time of treatment) probably failed treatment or were reinfected. These patients should be retreated and reevaluated for HIV infection. Because treatment failure usually cannot be reliably distinguished from reinfection with* T. pallidum*, CSF studies also should be performed.

Again, although failure of nontreponemal test titers to decline fourfold within 6–12 months after therapy for primary or secondary syphilis might be indicative of treatment failure, persons whose titers do not decline should be reevaluated for HIV infection. Optimum management of such patients is unclear. At a maximum, these patients should receive additional clinical and serologic follow up. If additional follow-up cannot be ensured, retreatment is recommended.

As a rule, a CSF examination should be performed in all patients with (1) serological evidence of syphilis and neurological symptoms and (2) when serological titers among asymptomatic patients do not respond appropriately to recommended therapeutic regimens. In other words, per current CDC recommendations, CSF examination should be performed in all asymptomatic patients with early syphilis whose RPR titers fail to demonstrate a 4-fold or greater decline 6–12 months after therapy, in patients with late latent syphilis whose RPR titers fail to demonstrate a similar decline 12–24 months after therapy, or in any stage of patient whose titers increase 4-fold or greater after therapy [5].

If CSF pleocytosis was present initially, a CSF examination should be repeated every 6 months until the cell count is normal. Follow-up CSF examinations also can be used to evaluate changes in the CSF VDRL or CSF protein after therapy; however, changes in these two parameters occur more slowly than cell counts, and persistent abnormalities might be less important. The leukocyte count is a sensitive measure of the effectiveness of therapy. If the cell count has not decreased after 6 months or if the CSF cells count or protein is not normal after 2 years, retreatment should be considered according to CDC guidelines.

## 7. Follow-Up of Neurosyphilis Cases: Possible without Lumbar Puncture?

Success of neurosyphilis treatment is defined by normalization of CSF and clinical abnormalities [6]. For patients who are neurologically asymptomatic, assessment of the efficacy of neurosyphilis treatment relies solely on normalization of CSF measures by repeated lumbar punctures. For those with symptoms or signs, resolution of clinical abnormalities is also considered when determining the efficacy of treatment. However, patients who have received treatment of neurosyphilis may be reluctant to undergo repeated lumbar puncture to assess the effectiveness of therapy. In a longitudinal cohort study, in patients treated specifically for neurosyphilis, the positive predictive value of normalization of serum RPR titer for normalization of CSF WBC count, CSF protein concentration, and CSF VDRL titer and resolution of meningitis and ocular disease by 4, 7, and 13 months after treatment of neurosyphilis were determined. Normalization of serum RPR titer predicted normalization of all measures except CSF protein concentration with a high degree of certainty. Except for the odds of resolution of eye disease which could not be determined because of insufficient data, the odds of normalization of the remaining measures were significantly higher when serum RPR titer had normalized than they were when serum RPR titer had not normalized (~28-fold higher for CSF protein concentration and meningitis, ~42-fold higher for CSF WBC count, and approximately 57 fold higher for CSF VDRL titer). Importantly, the odds of normalization of CSF WBC count and CSF VDRL titer were significantly lower when the pretreatment serum RPR titer was >1 : 32 [4].

## 8. How to Treat Syphilitic Meningitis

On the basis of lumbar puncture and duration of clinical manifestations, a decision could be made whether it is a case of syphilitic meningitis or relapsed case of primary or secondary syphilis.

For retreatment of relapsed syphilis (primary, secondary, or latent), weekly injections of benzathine penicillin G 2.4 million units IM for 3 weeks are recommended, unless CSF examination indicates that neurosyphilis is present [5]. If CSF examination indicates neurosyphilis, the recommended regimen is aqueous crystalline penicillin G, 18–24 million units per day, administered as 3-4 million units IV every 4 hours or continuous infusion, for 10–14 days. There remains a need for effective alternatives to PCN, and indeed, some alternative treatments for neurosyphilis have been discussed [7, 8]. For example, the prolonged half-life, adequate CNS penetration, and adequate activity against* T. pallidum* of ceftriaxone suggest that this drug may be an appropriate candidate for consideration. Doxycycline, tetracycline, amoxicillin, and probenecid are different treatment regimens of neurosyphilis used in the UK [7]. However no new therapeutic options including ceftriaxone have emerged to equal or supplant the standard recommended regimens of PCN [8]. Although azithromycin has not been used in the treatment of neurosyphilis, its use in the treatment of primary and latent syphilis is controversial. Recent reports of azithromycin-resistant* Treponema pallidum* in the United States indicate the importance of continued monitoring for resistance [9] (Table 2).

## 9. Conclusion

The current view is that a fourfold decline in lipoidal titers is sufficient to define cure, even if the lipoidal titers remain positive thereafter. However, this may not be true in every case of syphilis. As in this case of early symptomatic neurosyphilis, we see that even though RPR titers declined more than fourfold, neurosyphilis was nevertheless present and the diagnosis was missed. So even without headache or any neurologic signs or symptoms, if baseline serum RPR titers are more than 1 : 32 with evidence of syphilitic dissemination, when there is no other obvious source of active disease besides syphilis to explain a high RPR titer, a CSF analysis should be added in the recommendation of neurosyphilis diagnosis or to exclude neurosyphilis, especially with the fact that neurosyphilis, asymptomatic, or symptomatic is curable. Without being treated asymptomatic neurosyphilis can progress to symptomatic early or late neurosyphilis. Any RPR titer > 1 : 32 is highly suggestive of diseases of an active case of replicating spirochetes [2].

## Figures and Tables

**Table 1:** Classifications of neurosyphilis [2].

Manifestation	Percentage of cases (*N* = 676)
Syphilitic meningitis as a complication of secondary syphilis	8–40
Asymptomatic	7–38
Symptomatic	1-2
Asymptomatic late neurosyphilis	31
Symptomatic late neurosyphilis	69
Meningovascular	
Cerebromeningeal	6
Diffuse	
Focal	
Cerebrovascular	10
Spinal	3
Parenchymatous	
Tabetic	30
Paretic	12
Taboparetic	3
Ocular	3
Miscellaneous	2

Adapted from [10].

**Table 2:** Treatment of syphilis [3].

Syphilis stage or diagnosis	Primary therapy	Alternative therapy	Comment
Primary, secondary, and early latent syphilis	Penicillin G benzathine, 2.4 million units IM as a single dose	Doxycycline, 100 mg PO twice daily for 14 d or ceftriaxone, 1-2 g either IM or IV daily for 10–14 d or tetracycline, 100 mg PO four times daily for 14 d	—

Late latent syphilis	Penicillin G benzathine, 2.4 million units IM once weekly for 3 wk	Doxycycline, 100 mg PO twice daily for 28 d or tetracycline, 100 mg PO four times daily for 28 d	—

Neurosyphilis	Penicillin G aqueous, 18–24 million units IV daily (3-4 million units q 4 h or by continuous infusion) for 10–14 d	Procaine penicillin, 2.4 million units IM daily plus probenecid, 500 mg PO four times daily, both for 10–14 d or ceftriaxone, 2 g either IM or IV daily for 10–14 d	Follow-up treatment with 3 additional weekly injections of penicillin G benzathine, 2.4 million units IM

Tertiary syphilis (not neurosyphilis)	Penicillin G benzathine, 2.4 million units IM once weekly for 3 wk	—	Cerebrospinal fluid evaluation should be performed before therapy
